# Human *NR5A1*/SF-1 Mutations Show Decreased Activity on BDNF (Brain-Derived Neurotrophic Factor), an Important Regulator of Energy Balance: Testing Impact of Novel SF-1 Mutations Beyond Steroidogenesis

**DOI:** 10.1371/journal.pone.0104838

**Published:** 2014-08-14

**Authors:** Jana Malikova, Núria Camats, Mónica Fernández-Cancio, Karen Heath, Isabel González, María Caimarí, Miguel del Campo, Marian Albisu, Stanislava Kolouskova, Laura Audí, Christa E. Flück

**Affiliations:** 1 Department of Pediatrics, 2nd Faculty of Medicine, Charles University in Prague and University Hospital Motol, Prague, Czech Republic; 2 Pediatric Endocrinology, Department of Pediatrics and Department of Clinical Research, University Children’s Hospital Bern, Bern, Switzerland; 3 Pediatric Endocrinology, Vall d’Hebron Research Institute VHIR, CIBERER, Autonomous University, Barcelona, Spain; 4 Institute of Medical and Molecular Genetics INGEMM, Hospital Universitario La Paz, Universidad Autónoma de Madrid, IdiPAZ, Madrid, Spain; 5 Pediatric Endocrinology Service, Hospital Universitario La Paz, Universidad Autónoma de Madrid, IdiPAZ, Madrid, Spain; 6 Pediatric Endocrinology, Son Espases University Hospital, Palma de Mallorca, Spain; 7 Genetic Service, Hospital Vall d’Hebron, Barcelona, Spain; University of Louisville, United States of America

## Abstract

**Context:**

Human *NR5A1*/SF-1 mutations cause 46,XY disorder of sex development (DSD) with broad phenotypic variability, and rarely cause adrenal insufficiency although SF-1 is an important transcription factor for many genes involved in steroidogenesis. In addition, the Sf-1 knockout mouse develops obesity with age. Obesity might be mediated through Sf-1 regulating activity of brain-derived neurotrophic factor (BDNF), an important regulator of energy balance in the ventromedial hypothalamus.

**Objective:**

To characterize novel SF-1 gene variants in 4 families, clinical, genetic and functional studies were performed with respect to steroidogenesis and energy balance.

**Patients:**

5 patients with 46,XY DSD were found to harbor *NR5A1*/SF-1 mutations including 2 novel variations. One patient harboring a novel mutation also suffered from adrenal insufficiency.

**Methods:**

SF-1 mutations were studied in cell systems (HEK293, JEG3) for impact on transcription of genes involved in steroidogenesis (*CYP11A1*, *CYP17A1*, *HSD3B2*) and in energy balance (*BDNF*). BDNF regulation by SF-1 was studied by promoter assays (JEG3).

**Results:**

Two novel *NR5A1*/SF-1 mutations (Glu7Stop, His408Profs*159) were confirmed. Glu7Stop is the 4^th^ reported SF-1 mutation causing DSD and adrenal insufficiency. *In vitro* studies revealed that transcription of the *BDNF* gene is regulated by SF-1, and that mutant SF-1 decreased *BDNF* promoter activation (similar to steroid enzyme promoters). However, clinical data from 16 subjects carrying SF-1 mutations showed normal birth weight and BMI.

**Conclusions:**

Glu7Stop and His408Profs*159 are novel SF-1 mutations identified in patients with 46,XY DSD and adrenal insufficiency (Glu7Stop). *In vitro*, SF-1 mutations affect not only steroidogenesis but also transcription of BDNF which is involved in energy balance. However, in contrast to mice, consequences on weight were not found in humans with SF-1 mutations.

## Introduction

The nuclear receptor steroidogenic factor 1 (SF-1/*NR5A1*) is a master regulator of adrenal and gonadal development, including sexual determination and differentiation, as well as steroidogenesis and reproduction [1], [2]. SF-1 also plays a pivotal role in the development of the ventromedial hypothalamic nucleus (VMH) and for functions of the pituitary gland [3], [4]. In addition, expression of SF-1 has been identified in the spleen, skin and in small amounts in the placenta [5], [6], [7]. SF-1 was first identified in 1992 for its function as a transcriptional regulator of steroidogenic genes and was named accordingly [8]. Later, the Sf-1 KO mice were reported with a severe phenotype including lack of adrenal glands and a complete sex reversal of 46,XY animals [9]. Finally in 1999, the first human being with adrenal insufficiency and 46,XY disorder of sexual development (DSD) harboring a heterozygote SF-1 mutation was described [10]. Meanwhile, numerous SF-1 mutations have been identified [2], yet the exact function of SF-1 explaining the broad phenotype associated with SF-1 mutations remains elusive [11], [12].

SF-1 is encoded by the *NR5A1* gene which is located on the long arm of chromosome 9 (9q33). The gene consists of 7 exons but only 6 exons are coding. SF-1 has 461 amino acids and comprises a DNA binding domain with two zinc fingers, an accessory DNA binding domain, a hinge region and a ligand binding domain [1]. The structure of SF-1 is greatly conserved among animal species [13]. To date more than 70 human *NR5A1* mutations have been described (Human Gene Mutations Database, www.hgmd.cf.ac.uk). Most of these mutations are found in a heterozygote state [11], [14] and only a few in a homozygote [15], or compound heterozygote state [16]. So far, no correlation between phenotype and genotype, and also no clear pattern of heredity has been seen as both sporadic and familiar presentations exist [11]. Furthermore, possibility of dominant negative effect of heterozygote *NR5A1* mutations has been debated without convincing results [11].

The clinical presentation of SF-1 deficiency is very variable. The first human individual with a heterozygote Gly35Glu SF-1 mutation had 46,XY DSD and adrenal insufficiency [10]. So far, only two additional SF-1 mutations causing adrenal insufficiency have been reported [15], [17]. The heterozygote Arg255Leu mutation was found in a girl with symptoms of adrenal insufficiency and normal ovarian differentiation and function [17], and the homozygote Arg92Gln mutation was present in a boy with adrenal failure and 46,XY DSD [15]. By contrast, *NR5A1* mutations are frequently found in patients with 46,XY DSD with apparently normal function of the adrenal cortex [11], [14], [18]. Similarly, some *NR5A1* mutations were found in 46,XX females with premature ovarian failure or ovarian insufficiency with normal adrenal function [11], [19].

SF-1 deficiency also affects the central regulation of reproduction and energy balance [20]. The pituitary Sf-1 KO mouse model showed that SF-1 is an essential regulator of gonadotropin (LH, FSH) expression [4], [21], [22]. These mice present with hypogonadotropic hypogonadism reflected by sexual immaturity, low weight of gonads and sterility [4]. Apart from the pituitary gland, SF-1 is also required for the development, organization and function of the ventromedial hypothalamus (VMH) [3], [23]. Mouse models have shown that a loss of SF-1 stimulation leads to disorganization of the VMH, thereby impairing its function related to anxiety, thermoregulation, sexual behavior and energy balance [24]. Selective deletion of Sf-1 in the VMH in mice prenatally resulted in late onset obesity [25], while the same deletion postnatally led to diet induced obesity and deregulated thermogenesis [25]. However, these possible effects of SF-1 deficiency have not yet been studied in humans harboring SF-1 mutations.

In this context, the brain-derived neurotrophic factor (BDNF) is an important regulator of energy balance [26]. It is a highly conserved neurotrophin which is thought to be a SF-1 target gene [27]. BDNF is expressed in several appetite-regulating centers in the hypothalamus and the hindbrain in both mouse and human [28]. Depletion of *Bdnf* or its receptor (TrkB) in mice results in excessive feeding, weight gain and features accompanied by the metabolic syndrome [26], [29]. Abnormal locomotor activity and late-onset obesity was also observed in a *Bdnf* heterozygote knockout mouse model or when *Bdnf* was inactivated in the central nervous system [29], [30]. In addition, reduced expression of BDNF was described in association with obesity in the leptin receptor deficient mouse [31], the Alzheimer disease mouse [32] and the Sf-1 KO mouse [3]. In humans, two reports show a relationship between BDNF (locus 11p14) and obesity [33], [34]. Patients with WAGR syndrome (Wilm’s tumor, aniridia, genitourinary anomalies and mental retardation, OMIM 194072) with a heterozygous 11p14 deletion including the *BDNF* gene suffer all from childhood onset obesity, while WAGR syndrome patients without genetic anomalies including the *BDNF* gene have normal prevalence of obesity [33]. Additionally, a girl with obesity and impaired cognitive function who has only one functional copy of the *BDNF* gene has been described [34].

We hypothesize that human SF-1 mutations may affect metabolism and that this effect could be mediated in part through BDNF. Therefore, in this study we characterize novel *NR5A1* mutations, one being associated with the rare phenotype of adrenal insufficiency and 46,XY DSD. We describe the effect of these SF-1 mutations *in* *vitro* on transcription of genes involved in steroidogenesis and on the *BDNF* gene which is important for central regulation of food intake. Finally, we describe some weight related parameters in our small cohort of *NR5A1* patients.

## Patients and Methods

### Patients and ethical approval

**Figure 1:**
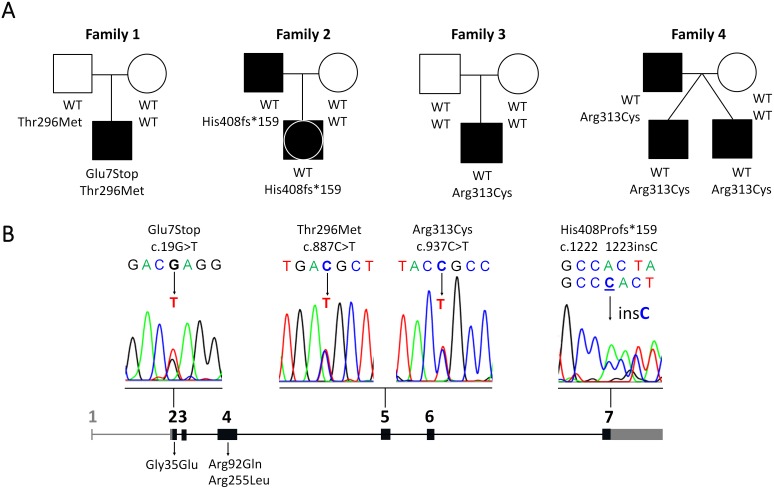
Genetic information on 5 subjects carrying *NR5A1* mutations. A. Family trees of 4 families and 7 affected individuals (5 patients and 2 parents) are shown. Scheme of the *NR5A1* gene showing the mutations identified in the reported patients (above the scheme) and all reported SF-1 mutations causing adrenal insufficiency (below the scheme). Electropherograms of novel mutations are also depicted. The *NR5A1* gene is composed of coding (*black*) and non-coding sequences (*gray*). Exons are indicated by *numbers*.

Five patients of Czech Republic and Spanish origin with unsolved 46,XY DSD were studied. Main characteristics of patients and families are shown in Table 1, family trees are depicted in Figure 1A and biochemical data are available as Table S1. All studied subjects and/or their legal guardians gave written informed consent for the hormonal and molecular studies, which were approved by the respective ethical committees of the involved centers: Ethics commissions of Vall d’Hebron Research Institute and CIBERER, Barcelona, Spain, and University Hospital Bern, Switzerland.

**Table 1:** Clinical and genetic characteristics of 5 patients harboring mutations in the *NR5A1* gene.

Patient	Origin,YOB	Karyotype	Assignedsex	Genital anatomyat birth	Gonadalfunction (age)	Adrenalfunction (age)	*NR5A1* genemutation	Familyhistory
1	Czech, 2010	46,XY	Male	Perineal hypospadia.Palpable testicles.	At 12 months: BaselineT low, FSH slightlyelevated.	Adrenal insufficiency(high ACTH, low cortisol,high PRA, normal aldosterone).	Compound heterozygote:c.19G>T, p.Glu7Stop;c.887C>T, p.Thr296Met	F = c.887C>T, p.Thr296Met
								M = WT
2	Spanish, 2013	46,XY	Male to femaleat 4 months	Clitoris with redundant skin.Palpable glans but no corpora cavernosa.Posterior labial fusion.Gonads palpable in genital folds (<1 ml).No Müllerian ducts.	Abnormal at 3.5 months:Baseline T low, FSH high;Very low T responseto hCG stimulation.	Normal at baselineaged 3.5 months.	Heterozygote: c.1222_1223insC, p.His408Profs*159	M = WT
								F = “carrier” (hypospadias)
								ICSI product from both parents’ gametes.
3	Spanish, 2011	46,XY	Male	Scrotal hypospadias.Penis length 2 cm.Bilateral scrotal gonads.No Müllerian ducts.	Abnormal: T slightly decreased,but normal precursor responseto hCG stimulation. AMHlow at age 17–20 days.	Normal at baselineaged 17 days.	Heterozygote:c.937C>T,p.Arg313Cys	F = WT
								M = WT
4	Spanish, 2010	46,XY	Male	Scrotal hypospadias. Penis length 1.5 cm.Bilateral scrotal gonads (1 ml).No Müllerian ducts.	T high at birth. AMH low at 2 months.	ND	Heterozygote:c.937C>T,p.Arg313Cys	Bichorial twin of patient 5.
	1st twin							F = carrier (operated hypospadias; testis volume L 6 ml/R 12 ml).
								M = unknown
								ICSI product from donor ova and father’s sperm.
5	Spanish, 2010	46,XY	Male	Scrotal hypospadias.Penis length 1.5 cm.Unilateral cryptorchidism,scrotal testis 0.5 ml.	T high at birth.AMH low at 2 months.	ND	Heterozygote:c.937C>T,p.Arg313Cys	Bichorial twin of patient 4.
	2nd twin							

YOB: year of birth; F: father; M: mother; WT: wild type; L: left; R: right; ND – not determined.

### Case reports (Table 1 and 2, Figure 1, Table S1)

Patient 1 from Czech Republic, was delivered at 31 weeks gestation because of HELLP (hemolysis, elevated liver enzymes, low platelets) syndrome of the mother. Birth weight was 1430 g (5–10^th^ percentile) and length was 38 cm (5–10^th^ percentile). Physical exam revealed perineal hypospadias but no other anomalies. Karyotype was 46,XY. Neonatal period was unremarkable. However, at the age of three months, he was admitted for adrenal failure (hyponatremia, hyperkalemia, episode of hypoglycemia, dehydration) in the course of an acute, viral respiratory infection. Baseline levels of ACTH and plasma renin activity were elevated. Cortisol response to ACTH stimulation was low confirming adrenal insufficiency.

Patient 2 from Spain (with parents from Argentina of European descents) was investigated during fetal development because of a discordant genital sex by ultrasound (female) compared to the genetic sex (46,XY). He was conceived by ICSI from both parents gametes. Owing to the father‘s history of hypospadias, the *NR5A1* gene was analysed in the father showing an heterozygous mutation. The same mutation was detected in fetal material. Although there was no consanguinity, the mother was also analysed to predict possible compound heterozygocity or homozygocity. The patient was delivered at 40 weeks gestation with a normal weight and length. External genitalia showed a clitoris-like genital tubercle, posterior labial fusion, no visible urethral meatus nor vaginal opening. Small (<1 ml) gonads were palpable in the genital folds. Male sex was assigned. The neonatal period was uneventful and endocrine evaluation was not performed until the age of 3.5 months when baseline ACTH, cortisol and 17-hydroxy-progesterone (17OHP) were normal, while baseline testosterone (T) was low and FSH high for age; T response to hCG stimulation was low. Therefore, at 4 months of age, gender was reassigned to female due to the severely feminized external genitalia.

Patient 3 from Spain was delivered at 38 weeks gestation with normal weight and length; he was evaluated at 14 days due to ambiguous genitalia (scrotal hypospadias, penis length 2 cm and gonads palpable in the scrotal folds). Karyotype was 46,XY. Baseline cortisol and aldosterone were normal as were the measured steroid precursors. Baseline FSH was slightly elevated, AMH and inhibin B were low. T response to hCG stimulation was low while the T/DHT ratio was normal.

Patients 4 and 5 from Spain were delivered as bichorionic twins at 36 weeks gestation with the 1st twin showing normal weight and lenght while the 2nd was small for gestational age (SGA). They were obtained by ICSI from the father’s sperm and a donated ova to avoid the mother’s genetic disease (epidermiolysis bullosa). Both babies presented with ambiguous genitalia: scrotal hypospadias with a penis length of 1.5 cm, both gonads palpable (1 ml) in the scrotal folds (1st twin) and unilateral cryptorchidism and one palpable gonad (0.5 ml in the 2nd twin). The father had been operated for hypospadias during childhood. His testes biopsies at 9 years of age revealed diminished seminiferous tubule diameter and fertility index in the left testis while these parameters were normal in the right testis). Neonatal period was uneventful in both twins. At birth (2 days), baseline T, precursors and gonadotropins were normal for age and AMH was low at 2 months.

### Genetic analyses

Genomic DNA was isolated from peripheral blood leukocytes. All exons and part of adjacent introns of the *NR5A1* gene were amplified and sequenced as previously described [11]. Obtained sequences were analysed against the *NR5A1*/SF-1 GenBank entries NT_008470.19 (genomic DNA), NM_004959.4 (mRNA) and NP_004950.2 (protein).

### In vitro functional studies

Human embryonic kidney cells (HEK293) and human placental choriocarcinoma cells (JEG3) were used for functional studies. HEK293 cells were cultured in DMEM supplemented with 10% fetal calf serum, 1% penicillin/streptomycin and 1% sodium pyruvate (Gibco, Paisley, UK). JEG3 cells were cultured in MEM supplemented with 10% fetal calf serum, 1% penicillin/streptomycin and 1% L-glutamine (Gibco).

Promoter luciferase reporter vectors for steroidogenic enzymes HSD3B2, CYP11A1, CYP17A1 (-227CYP17A1_Δluc, -152CYP11A1_pGL3, -301HSD3B2_pGL3) and corresponding empty control vectors (Δ_luc, pGL3), as well as cDNA for wild-type (WT) SF-1/NR5A1 were available from previous work [35], [36]. Luciferase reporter vectors for human promoters I, IV, V and VII of the *BDNF* gene (pGL4.15_hBDNFpI, pGL4.15_hBDNFpIV, pGL4.15_hBDNFpV, pGL4.15_hBDNFpVII) were kindly provided by Dr. P. Pruunsild and Prof. T. Timmusk (Tallinn University of Technology, Tallinn, Estonia). Mutant NR5A1 expression vectors (c.19T, c.887T, c.937T, c.1222_1223insC) and SF-1 cis element mutant pGL4.15_hBDNFpI (c.-876_873CTTT) were generated by PCR-based site-directed mutagenesis using specific primers (available upon request) following the QuickChange protocol by Stratagene (Agilent Technologies Inc., Santa Clara, CA, USA).

For promoter activity studies, cells were cultured in 24-well plates and transiently transfected with WT or mutant NR5A1 and WT or mutant promoter luciferase reporter constructs using Lipofectamine 2000 (Invitrogen, AG, Basel, Switzerland) for 48 hours, and the Dual-Luciferase Reporter (DLR) Assay System (Promega AG, Wallisellen, Switzerland) was used for readout as previously described [11]. Specific *Firefly* luciferase readings were normalized against *Renilla* control readings and expressed as relative light units (RLU). Experiments were repeated at least 3 times in duplicates.

### Statistical analysis

Data are shown as mean±SEM of at least three independent experiments. Statistical analysis was examined by t-test with Microsoft Office Excel (Windows 2003, Microsoft Inc.). Significance was set at *p<0.05, **p<0.01.

## Results

### Genetic analysis

We identified 2 novel and 2 known *NR5A1* sequence variations in 5 patients newly diagnosed with SF-1 deficiency (Figure 1). Patient 1 (family 1), a boy with adrenal insufficiency and 46,XY DSD harbored compound heterozygote novel c.19G>T and c.887C>T variations in the *NR5A1* gene coding for Glu7Stop in the DNA binding domain and Thr296Met in the ligand binding domain. Glu7Stop was not found in either parents, thus qualifying as a *de* *novo* mutation. By contrast, heterozygocity for Thr296Met was detected in the healthy father (Figure 1). Patient 2 (family 2) was heterozygote for a novel insertion c.1222_1223insC which is predicted to result in a frameshift elongating the SF-1 protein by 159 amino acids (His408Profs*159). The mutation was inherited from an affected carrier father (Figure 1). In patients 3 to 5 from two families (3 and 4), a heterozygote c.937C>T mutation in exon 5 was found changing Arg313Cys in the ligand-binding domain (Table 1, Figure 1). In family 3, the mutation was *de* *novo* (patient 3), whereas in family 4 (patients 4 and 5), the mutation was inherited in two dizygotic twins from an affected carrier father (Table 1, Figure 1). This mutation was recently reported in a patient manifesting with distal hypospadias and a bifid scrotum containing testes [37].

### In vitro studies of the impact of SF-1 variants on steroidogenesis and energy balance

**Figure 2:**
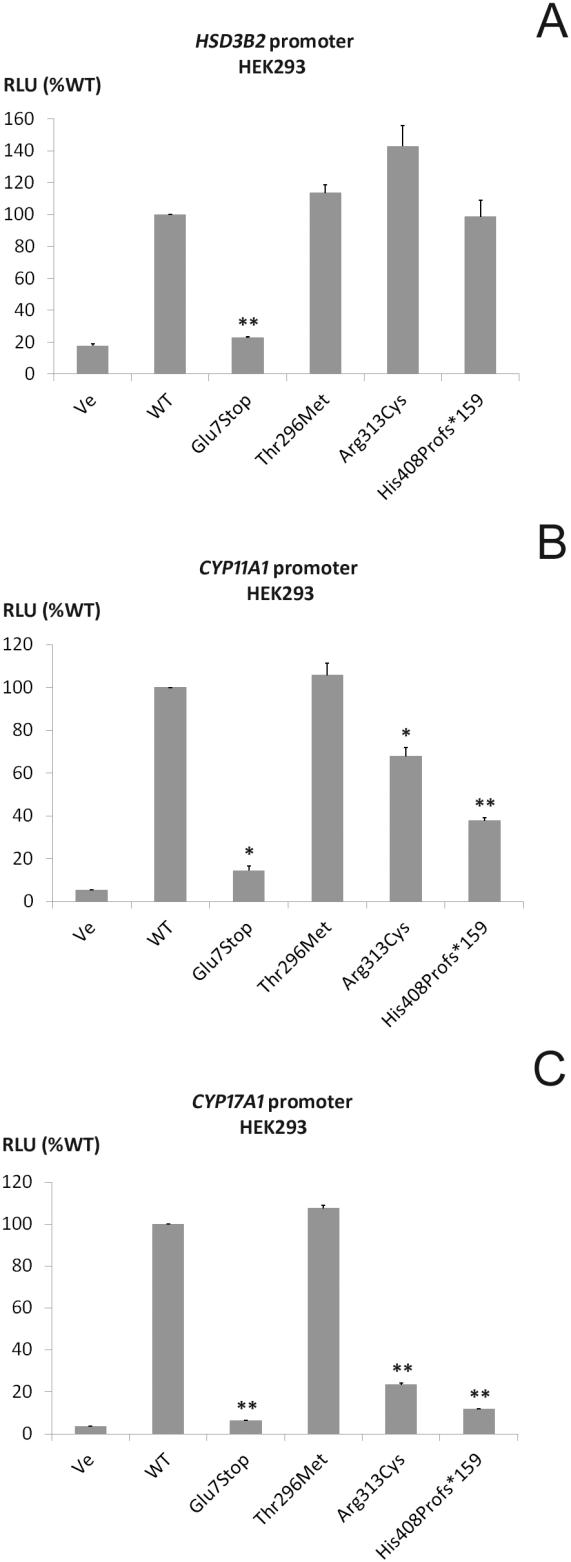
Promoter reporter studies for reported *NR5A1* mutations . Human embryonic kidney HEK293 cells were transiently transfected with wild-type (WT) or mutant SF-1 and promoter luciferase reporter constructs of the genes for steroidogenic enzymes *HSD3B2* (A), *CYP11A1* (B), *CYP17A1* (C). Luciferase activity was measured with the Promega Dual Luciferase assay system. Results are expressed as percentage of WT SF-1 activity. Independent experiments were performed in duplicate at least 3 times. Error bars represent the mean and SEM. *p<0.05; **p<0.01.

Impact of identified SF-1 mutations on steroidogenesis was studied in non-steroidogenic HEK293 cells by assessing their transcriptional activity on the promoters of the *HSD3B2*, *CYP11A1* and *CYP17A1* genes (Figure 2). The Glu7Stop mutation revealed a complete lack of transcriptional activation for all promoter constructs. The Thr296Met variant showed similar transactivation activity as WT SF-1 indicating that it is not a disease-causing variant but rather a polymorphism (SNP). Interestingly, the His408Profs*159 and Arg313Cys mutations showed normal activity when tested on the *HSD3B2* promoter construct, but their transactivation activity was significantly decreased when tested on the *CYP11A1* and *CYP17A1* promoters.

**Figure 3:**
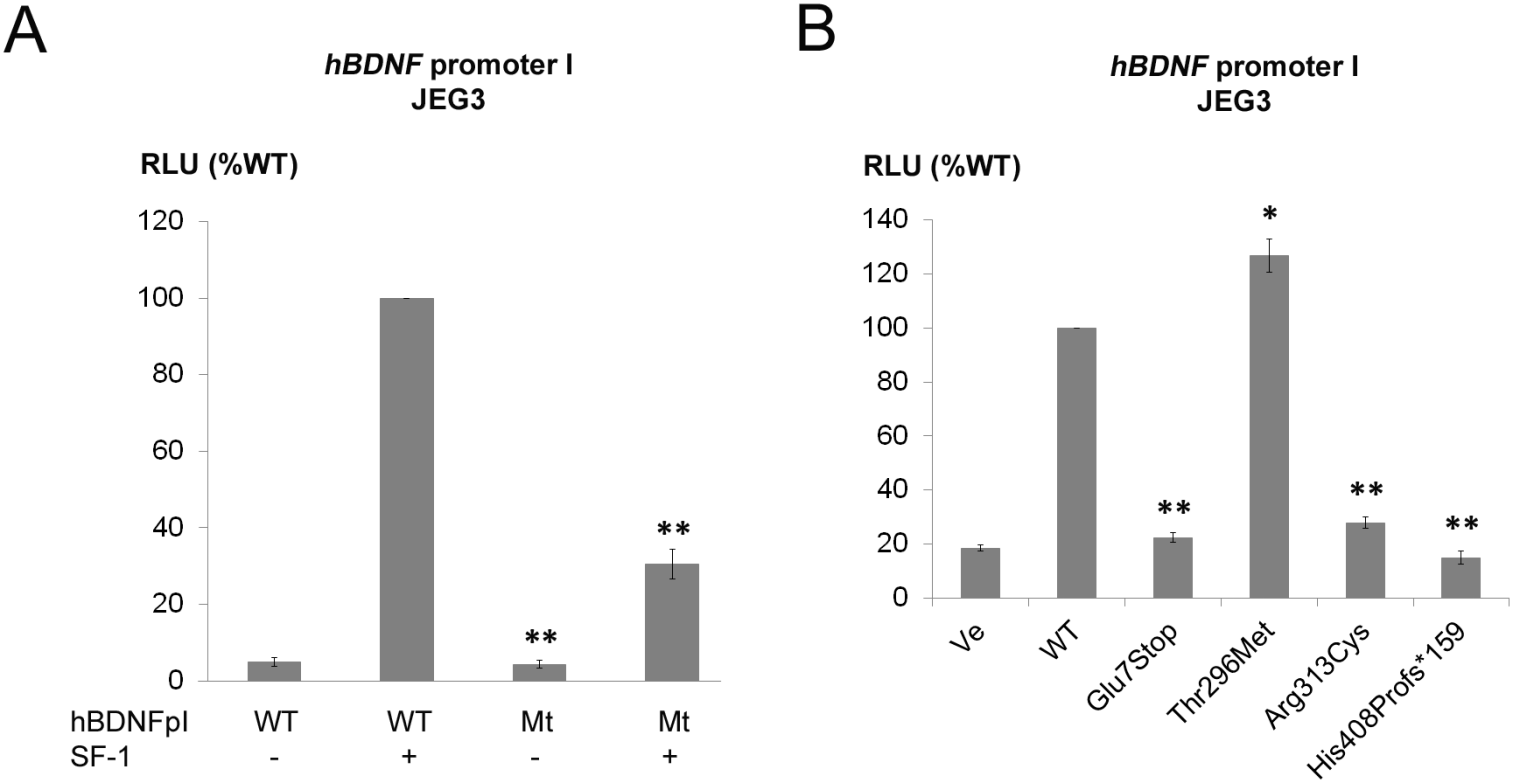
Promoter reporter studies for SF-1 regulating the human *BDNF* promoter I and comparative studies of specific *NR5A1* mutations on *BDNF* promoter activity. A consensus SF-1 transcription binding site was identified in the wild-type (WT) human BDNF promoter I. This *cis*-element was mutated to assess the role of SF-1 on BDNF transcription. Human placental JEG3 cells were transiently transfected with the WT or the SF-1 element mutant (Mt) BDNF promoter reporter with or without SF-1, and promoter activity was assessed by the Promega Dual Luciferase assay (A). After showing that SF-1 regulates the WT BDNF promoter I specifically (A), the ability of specific SF-1 mutations to *trans*-activate the *BDNF* promoter I was investigated (B). Results are expressed as percentage of WT SF-1 activity. Independent experiments were performed in duplicate 3 times. Error bars represent the mean and SEM. *p<0.05; **p<0.01.

To study potential involvement of SF-1 on central energy balance, the transcriptional regulation of SF-1 on the promoters of the *BDNF* gene was assessed in JEG3 cells. According to literature, there are many alternative BDNF variants (17 BDNF and 12 antisense BDNF variants) in human due to use of different promoters [28]. For initial experiments, we used four different human *BDNF* promoter constructs, namely *hBDNF* I, IV, V and VII. Promoter constructs I and IV were chosen as mouse *Bdnf* transcripts I and IV are primarily expressed in the brain (area of VMH) and their promoters are reported to be regulated by Sf-1 [27]. Similarly, promoters V and VII were assessed for reported expression in the human brain [28]. In our JEG3 cell system, among those promoters, we were only able to transactivate the *hBDNF* promoter I by WT SF-1 (data not shown). Thus, further studies involving SF-1 and human BDNF were performed with this *hBDNF* promoter I (Figure 3). In this promoter, we found a putative SF-1 *cis*-element at c.-874 to -867 (CAAGGACA). To confirm that this cis-element in the *hBDNF* promoter is regulated by SF-1, we constructed a promoter with a mutant SF-1 site and assessed its activity by co-transfection with WT SF-1. Upon co-transfection with WT SF-1, the mutant *BDNF* promoter lost activity when compared with the WT promoter (Figure 3A). In this system, SF-1 mutants Glu7Stop, Arg313Cys and His408Profs*159 showed only weak transactivation activity compared to WT SF-1 confirming a possible effect of SF-1 on BDNF and thus energy balance. By contrast, the Thr296Met sequence variation had similar transactivation power on the *hBNDF* promoter I as seen with WT SF-1 (Figure 3B).

### Weight related parameters of patients harboring SF-1 mutations

To address the question whether SF-1 deficiency may have metabolic consequences, we collected clinical data from our cohort of patients with SF-1 mutations. We were able to obtain data from 16 subjects with heterozygote SF-1 mutations including patients and their (affected) relatives (Table 2). Birth weight in singletons was normal (n = 8; median −0.83 SD, range −1.9 to 1.21). BMI of subjects currently being 1–17 years of age was also normal (n = 9; median 0.04 SD, range −0.69 to 2.81), as was BMI of 5 adults (median 0.6 SD, range 0.4 to 0.84). Thus in our small cohort of rather young patients with heterozygote SF-1 mutations overweight or obesity seems not an issue.

**Table 2:** Weight related parameters of subjects carrying (heterozygote) *NR5A1* mutations.

Subject	Karyotype	*NR5A1* mutation(s)		Gestationalage at birth	Birth weight		Current age	Actual weight			BMI	
				(weeks)	(g)	(SD)	(y, m)	(kg)	(SD)		(kg/m^2^)	(SD)
*Present study* *(* *Figure 1* *)*												
F1, II.1	46,XY	Glu7Stop;Thr296Met	31		1430	−0.83	3 y 1 m	16.30		0.59	17.80	1.39
F2, II.1	46,XY	His408Profs*159	40		3200	−0.76	-	-		-	-	-
F3, II.1	46,XY	Arg313Cys	38		2630	−1.77	-	-		-	-	-
F4, II.1	46,XY	Arg313Cys	36		2470	−0.75	1 y 10 m	12.70		0.30	15.30	−0.26
F4, II.2	46,XY	Arg313Cys	36		1390	−4.87	1 y 10 m	13.00		0.57	15.40	−0.47
F2, I.1_Fa	46,XY	His408Profs*159	-		-	-	32 y	80.00		-	27.00	0.50
F4, I.1_Fa	46,XY	Arg313Cys	-		-	-	30 y	90.00		-	26.60	0.40
*Patients from Camats et al.* *(* *Table 1* *, Ref. 11)*												
1	46,XY	Val20Leu	41		2970	−1.90	5 y	21.80		0.80	15.30	0
2	46,XY	His24Tyr	40		3600	0	17 y	85.00		-	25.70	1.33
5	46,XY	Gly90Arg	40		3100	−1.28	14 y 6 m	51.40		0	19.70	0.04
6	46,XY	Pro130ArgfsX165	-		-	-	1 y	8.90		−1.15	15.90	−0.69
7	46,XY	Gln206ThrfsX20	41		3820	1.21	5 y	26.30		2.90	21.70	2.81
8	46,XY	Leu231_Leu233dup	40		3280	−0.67	5.5 y	28.00		2.85	21.40	2.45
9	46,XX	Pro235Leu	-		-	-	19 y	58.00		-	24.00	0.60
1_Fa	46,XY	Val20Leu	-		-	-	32 y	71.00		-	24.00	0.84
5_Mo	46,XX	Gly90Arg	-		-	-	35 y	63.00		-	23.40	0.76

F: family; Fa: father; Mo: mother.

## Discussion

In this study, two novel SF-1 mutations (Glu7Stop, His408Profs*159) were identified in 5 patients with SF-1 deficiency, all manifesting with 46,XY DSD and one (Glu7Stop) presenting with adrenal insufficiency that is rarely associated with SF-1 mutations. The disease causing impact of the identified mutations was confirmed by functional studies in cell models assessing transcriptional activity of wild-type and mutant SF-1 on genes involved in steroidogenesis (*CYP11A1, CYP17A1, HSD3B2*) and central energy balance (*BDNF*).

To date, only three SF-1 mutations have been implicated with adrenal insufficiency (heterozygote Gly35Glu and Arg255Leu, homozygote Arg92Gln) [10], [15], [17]. With patient 1, we add a novel SF-1 mutation (Glu7Stop) to this series. Our *in* *vitro* studies suggest that Glu7Stop is a loss of function SF-1 mutation. By contrast, the second SF-1 sequence variation (Thr296Met) identified in patient 1 is rather a simple polymorphism. First, it does not differ in functional assays when compared to WT SF-1. Second, this variant (rs201151141) has also been detected at an allelic frequency of 0.001 in a cohort of 662 normal subjects studied for a large-scale genome sequencing project (dbSNP database, http://www.ncbi.nlm.nih.gov/projects/SNP/snp).

The novel His408Profs*159 mutation identified in the SF-1 gene in patient 2 codes for a longer protein of 567 amino acids compared to WT SF-1 (461 aa). This mutant had an impact on both the steroidogenic promoters and the *BDNF* promoter (Figure 2). Interestingly, this heterozygous mutation was first detected in the father when investigated because of his history of childhood hypospadias and infertility. He nevertheless fathered a child through ICSI but the fetus’s genetic and phenotypic sex was discordant. At birth the 46,XY child’s genital phenotype was almost completely feminized and hormonal work-up at 4 months of age revealed low androgens prompting female sex assignment although the mutation was the same as in the father. This illustrates again for a novel *NR5A1* mutation the wide phenotypic spectrum within the same family.

The Arg313Cys mutation found in patients 3–5 in our study was recently reported in a patient with hypospadias [37]. Similar to our functional assays, Arg313Cys showed reduced transactivation on the promoters of the *AMH* and *CYP11A1* genes [37]. Although Arg313 is located in the highly conserved helix 5 of the ligand-binding domain, this same position has also been described for an Arg313His change (c.838G>A) in males with hypospadias [38], [39]. Thus, position Arg313 of SF-1 may be a hot spot for mutations. Interestingly, this mutation appeared *de* *novo* in our family 4, while in family 5, the mutation was transmitted by an affected father.

In theory, SF-1 mutations could also have metabolic consequences for affected patients. But so far, no clinical data existed on this topic. Observations from the Sf-1 KO mice models suggest that loss of SF-1 is associated with impaired energy balance and low temperature expenditure leading to late-onset type of obesity [25]. Deletion of *BDNF* gene was described in obese patients with WAGR syndrome [33]. Among many other factors regulating appetite, SF-1 together with BDNF are expressed in the VMH of mice. Tran *et al*. described a significantly decreased expression of the *Bdnf* gene in Sf-1 +/− KO mice [27]. They also identified two promoter variants of the murine *Bdnf* gene which were specifically used in the VMH and their activity was related to SF-1 dosage [27]. In the presented study, we tried to establish the role of SF-1 in the regulation of human BDNF, an important player for central energy balance and thus obesity [40]. In fact, our experiments now show that the human *BDNF* promoter I is regulated by SF-1, and that SF-1 mutations have impaired transactivation activity on this promoter similar to impaired effect on promoters regulating genes of steroidogenesis. These results indicate that SF-1 might be a co-regulator of energy balance and that mutations in SF-1 may therefore also lead to metabolic consequences (e.g. obesity) in humans.

In addition to the *in* *vitro* studies, we were also able to collect some clinical data related to energy balance in 16 patients with heterozygote SF-1 mutations (Table 2). However, in our small cohort of 9 patients aged 1–17 years and 5 adults, we did not find weights and BMIs consistent with overweight or obesity. Therefore, presented clinical data in humans are not in line with data found in mice [25], [26], although *in* *vitro* data in human and mouse are similar [25], [27]. These negative results might be ‘true’ negative results or may be explained by the following shortcomings of our presented study. First, small number of studied patients, which are in addition too young to observe metabolic consequences. Second, all patients are heterozygote for their SF-1 mutation and carry one wild-type SF-1 allele while most studied mice were Sf-1 complete KO. Further collaborative studies are therefore needed to gather clinical data of more patients and follow-up on a bigger cohort longitudinally.

## Supporting Information

Table S1 **Biochemical data of patients included in this study.** Values outside the age- and sex-specific reference range are given in **bold**. ^1^PRA: plasma renin activity; ^2^AMH: anti-Müllerian hormone; ^3^Synacthen (250 µg/1.73 m^2^ BSA); ^4^hCG: 600 IU/48 h×6; ^5^hCG: 1000 IU/24 h×3.
(DOC)
