# Supplementary material for: Human NR5A1/SF-1 Mutations Show Decreased Activity on BDNF (Brain-Derived Neurotrophic Factor), an Important Regulator of Energy Balance: Testing Impact of Novel SF-1 Mutations Beyond Steroidogenesis
Source: PLoS One. 2014 Aug 14;9(8):e104838. doi: 10.1371/journal.pone.0104838 (PMC4133263; doi:10.1371/journal.pone.0104838)
Supplement: Table S1 — Biochemical data of patients included in this study. Values outside the age- and sex-specific reference range are given in bold. 1PRA: plasma renin activity; 2AMH: anti-Müllerian hormone; 3Synacthen (250 µg/1.73 m2 BSA); 4hCG: 600 IU/48 h×6; 5hCG: 1000 IU/24 h×3. (DOC) [file pone.0104838.s001.doc]

**Table S1.** Biochemical data of patients included in this study.

| **Serum levels** | |  | **Patient 1** | **Patient 2** | **Patient 3** | **Patient 4** | **Patient 5** |
| --- | --- | --- | --- | --- | --- | --- | --- |
| Age at investigation |  |  | 3 months | 3.5 months | 17 days | 2 days/2 months* | 2 days/2 months* |
| ACTH | (pM/L) |  | **25.70** | 4.30 |  |  |  |
| PRA^1^ | (μg/L/h) |  | **7.40** |  |  |  |  |
| Aldosterone | (nM/L) |  | 0.11 |  | 0.42 |  |  |
| Cortisol | (mM/L) | Baseline | **132** | 233 | 226 |  |  |
|  |  | Stimulated^3^ | **324** |  |  |  |  |
| 17OH-pregnenolone | (nM/L) |  | 5.50 |  |  |  |  |
| DHEA | (nM/L) | Baseline | 3.81 |  | 2.39 |  |  |
|  |  | Stimulated^3^ |  |  |  |  |  |
| DHEA-S | (mM/L) |  | 0.28 |  |  | 0.97 | <0.38 |
| Progesterone | (nM/L) |  |  |  | 41.60 |  |  |
| 17OH-progesterone | (nM/L) |  |  | 3.85 | 10.60 | 30.00 | 5.10 |
| Androstendione | (nM/L) | Baseline | **3.68** | 0.35 | 8.50 |  |  |
|  |  | Stimulated |  |  | 6.4^5^ |  |  |
| Testosterone | (nM/L) | Baseline | 9.14 | **<0.30** | **4.10** | 11.00 | 15.20 |
|  |  | Stimulated^4,5^ |  | **2.42^4^** | **7.1^5^** |  |  |
| Dihydrotestosterone | (nM/L) | Baseline | **1.64** |  | 0.50 |  |  |
|  |  | Stimulated |  |  | 0.6^5^ |  |  |
| SHBG | (nM/L) |  |  | 83.30 |  |  |  |
| LH | (IU/L) |  |  | 2.70 | 8.60 | <0.1 | <0.1 |
| FSH | (IU/L) |  |  | **10.90** | **19.40** | 0.50 | 0.50 |
| AMH^2^ | (μg/L) |  |  |  | **16.30** | **10.4*** | **13.7*** |

^1^ PRA: plasma renin activity; ^2^ AMH: anti-Müllerian hormone; ^3^ Synacthen (250μg/1.73m^2^BSA); ^4^ hCG: 600 IU/48h x 6; ^5^ hCG: 1000 IU/24h x 3.

Note: Values outside the age- and sex-specific reference range are given in **bold**.
